# Laparoscopy in Liver Transplantation: The Future has Arrived

**DOI:** 10.1155/2012/148387

**Published:** 2012-08-07

**Authors:** Quirino Lai, Rafael S. Pinheiro, Giovanni B. Levi Sandri, Gabriele Spoletini, Fabio Melandro, Nicola Guglielmo, Marco Di Laudo, Fabrizio M. Frattaroli, Pasquale B. Berloco, Massimo Rossi

**Affiliations:** ^1^Department of General Surgery and Organ Transplantation, Sapienza University of Rome, Umberto I Policlinic of Rome, Viale del Policlinico 155, 00161 Rome, Italy; ^2^Department of Liver Transplantation, University of São Paulo, 01005 010 São Paulo, SP, Brazil

## Abstract

In the last two decades, laparoscopy has revolutionized the field of surgery. Many procedures previously performed with an open access are now routinely carried out with the laparoscopic approach. Several advantages are associated with laparoscopic surgery compared to open procedures: reduced pain due to smaller incisions and hemorrhaging, shorter hospital length of stay, and a lower incidence of wound infections. Liver transplantation (LT) brought a radical change in life expectancy of patients with hepatic end-stage disease. Today, LT represents the standard of care for more than fifty hepatic pathologies, with excellent results in terms of survival. Surely, with laparoscopy and LT being one of the most continuously evolving challenges in medicine, their recent combination has represented an astonishing scientific progress. The intent of the present paper is to underline the current role of diagnostic and therapeutic laparoscopy in patients waiting for LT, in the living donor LT and in LT recipients.

## 1. Introduction

In the last decades, laparoscopy has revolutionized the field of surgery. Video laparoscopy was officially born in 1987, when Professor Phillipe Mouret performed the first cholecystectomy in Lyons, France [1]. Many procedures previously performed with the open technique are now carried out with the laparoscopic approach. Several advantages are associated with laparoscopic surgery compared to open procedures: reduced pain due to smaller incisions and haemorrhaging, shorter hospital length of stay, and a lower incidence of wound infections are all arguments that gave strength to the widespread of laparoscopy.

Similarly, liver transplantation (LT) has radically changed the care for many patients with hepatic end-stage diseases. The first human LT was performed in 1963 by Professor Thomas Starzl [2] in Denver, United States: however, due to its initial poor results, LT remained an experimental therapy for several years. Only introduction of cyclosporine [3] markedly improved patient outcomes, turning LT to a standard clinical treatment for more than fifty adult and paediatric liver pathologies and, at the same time, allowing to achieve excellent results in terms of survival. 

The intent of the present paper is to underline the current role of diagnostic and therapeutic laparoscopy in patients waiting for LT, in the living donor LT and in LT recipients.

## 2. Pretransplant Surgery

### 2.1. Laparoscopic Liver Resection

The first nonanatomical laparoscopic hepatectomy was performed by Gagner in 1992 [4] and the first anatomical one by Azagra in 1996 [5]. Since these first experiences, laparoscopic approach for hepatic resection has been adopted in different centres, showing its feasibility and safety in well-selected patients [6–10] and also confirming its prerogatives (shorter operative times, less bleeding) even in this very complex type of surgery [7, 11].

However, malignant tumors were initially considered a contraindication for mini-invasive approach. Only in the last years, this risk has been reconsidered, showing no difference in margin-free resection, port-site recurrence, or tumour seeding rates between open and laparoscopic techniques [12–15]. Consequently, malignant tumors do not represent anymore a contraindication for an expert surgeon in choosing a laparoscopic approach [16]. Until now, more than 3,000 minimally invasive hepatic resections have been reported in the literature [17], and-small-to medium sized procedures have become commonplace in many centres [18]. 

Recently, a consensus conference [19] has underlined that acceptable indications for laparoscopic liver resection are (a) solitary lesions with a diameter ≤5 cm, located in segments II–VI; (b) laparoscopic approach to left lateral sectionectomy (LLS) should be considered as a standard practice; (c) although all types of liver resection can be run laparoscopically, major liver resections (e.g., right or left hepatectomies) should be reserved for experienced surgeons facile with more advanced laparoscopic hepatic resections. 

The main indication for resection in patients waiting for LT is represented by hepatocellular carcinoma (HCC). Transplant surgeons are well aware that a LT after previous surgery could represent a real challenge, increasing technical difficulty caused by adhesions [20–22]. Basing on these considerations, laparoscopy as bridge to LT may reduce such problems [23]. 

Despite no prospective randomized controlled trials have been performed yet, some studies based on matched comparisons showed similar mortality and even lower morbidity rates after laparoscopy with respect to open liver resection. After laparoscopy, 3-year patient survival rates of 60–93% and 3-year disease-free survivals of 52–64% have been reported [8, 24–29]. 

Advent of robotic surgery has further improved the opportunity of mini-invasive treatment of HCC. The robotic approach may enable liver resection in patients with cirrhosis, allowing for technical refinements of laparoscopic liver resection due to 3-dimensional visualization of the operative field and instruments with wrist-type end-effectors [30–32].

### 2.2. Laparoscopic Radiofrequency Ablation

Radiofrequency (RF) ablation represents a nonsurgical locoregional treatment, used in very well-selected patients with nonresectable HCC waiting for LT. In the last years, laparoscopic or hand-assisted RF has shown promising necrosis and survival rates [33, 34], providing a substrate for their safe adoption [35]. The main advantage of laparoscopic RF with respect to percutaneous approach is the opportunity to detect pre-operatively undetectable lesions using an intraoperative ultrasound (IOUS) [36, 37]. IOUS remains the most sensitive imaging modality for HCC, being able to detect new lesions in 13.1% to 30% of cases [38–42]. In a study comparing laparoscopic liver resection and RF, Santambrogio et al. [43] identified 15 (20%) of 74 cases with previously undetected lesions in the RF group. Laparoscopic RF also consents to treat lesions considered inappropriate for percutaneous RF due to the high risk of injury in the diaphragm, stomach, or bowel [44–46]. Similarly, laparoscopic RF minimizes the risk of complications in patients previously operated in the upper abdominal quadrants [47].

### 2.3. Laparoscopic Kasai Procedure in Children with Biliary Atresia

Laparoscopic portoenterostomy, also named Kasai procedure, for biliary atresia was first reported by Esteves et al. in 2002 [48]. Besides its safety and feasibility, laparoscopic Kasai can provide the advantage of a lower hepatic adhesions rate, which ease the potential “salvage” LT. Martinez-Ferro et al. reported 41 cases of laparoscopic Kasai, with only one conversion [49], and encouraging results in terms of postoperative bile flow rates. However, the only prospective study comparing open and laparoscopic procedure was stopped after observing that laparoscopic patients showed a significantly shorter time between Kasai procedure and LT [50]. Therefore, the role of laparoscopic Kasai remains unclear [51], being reserved to paediatric centres with high specialization in minimally invasive surgery.

## 3. Laparoscopic Living Donor-Hepatectomy

The early idea of solid organ transplantation using living donors began with kidney transplants; similarly, laparoscopy for living donation was initially developed for kidney transplantation, with the intent to offer a less aggressive procedure to the donor. In fact, laparoscopic nephrectomy is associated with less postoperative pain, decreased length of hospital stay, faster return to normal activity, smaller scars, and less morbidity [52]. As a consequence, an increased number of kidney transplants using living donors have been recently observed in many centres [53, 54].

Living-donor liver transplantation is a complex procedure, with major risks of morbidity and mortality with respect to kidney donation: the reported mortality of this procedure has varied from 0.2% to 0.5% [55]. Clearly, donor risk increases according to the type of hepatectomy (LLS < left hepatectomy < right hepatectomy (RH)).

Typically, a LLS or a left hepatectomy is sufficient in a paediatric living liver donation, while a RH is necessary for an adult-to-adult donation. 

However, despite different surgical approaches could be adopted, open living donation always requires a large abdominal incision. This aspect, combined with postoperative pain, long hospital stay, and long periods of recovery, represents a barrier to donation, especially in young women [56].

Recently, experimental model has demonstrated the feasibility of laparoscopic living donation using the available technology [57].

Concurrent improvement in laparoscopic surgery for hepatic tumours enhanced feasibility and safety of more complex procedures [18], leading to the establishment of laparoscopic surgery for liver living donors. Soubrane et al. [58] reflected on the quality of the graft and the morbidity rates in the donor: they reported comparable results in both conventional and laparoscopic techniques apart from longer operative times and lower blood losses in the laparoscopy group. 

### 3.1. Paediatric Donation

Paediatric living donation provides similar or better short-term graft function and long-term survival rates with respect to postmortem donor LT: the first case of laparoscopic donation was reported by Cherqui et al. [56]. In 2006, Soubrane et al. [58] reported the safety of laparoscopic LLS in 16 consecutive live donors compared with the conventional LLS. According to the first series experienced, the liver graft typically includes the LLS (i.e., segments II and III according to Couinaud's classification), left branch of hepatic artery, left portal branch, left bile duct, and left hepatic vein. After these initial experiences, several other new series have been reported worldwide [59, 60]. 

Recently, Kim et al. compared 11 laparoscopic LLS for living donation with 11 open ones, showing that the laparoscopic group had significantly shorter hospital stay, whilst duration of operation, blood loss, warm ischemia time, and out-of-pocket medical costs were comparable between groups [61].

A similar study from Washington DC compared 15 laparoscopic or laparoscopic-assisted left or right hepatectomies for liver donation with 15 hepatectomies with open access: no substantial differences were observed in terms of early graft function, allograft biliary, and vascular complications and survivals (1-year graft and patient survival: 100% versus 93% in laparoscopic and open group, resp.) [62].

### 3.2. Adult Donation

In 2006, Koffron et al. [63] described the first hand-assisted laparoscopic RH for live donation. Kurosaki et al. [64] reported in the same period 13 consecutive video-assisted adult-to-adult laparoscopic hepatectomies (3 RH and 10 left ± segment I hepatectomies): surgical manipulation was obtained via ports or via a 12 cm incision whilst view resulted by a combination of direct and laparoscopic vision. Reported median operation time was 363 ± 33 minutes and a median blood loss of 302 ± 191 mL. No complications were reported, restoration of liver function was smooth, and analgesics use was inferior with respect to the historical control (median: 1.2 versus 3.8 times). 

In 2008, the transplant group from Seoul [65, 66] commented on the first series of hand-assisted laparoscopic modified RH preserving the middle hepatic vein; the authors reported 2 cases of laparoscopic RH and 7 cases of laparoscopy-assisted RH with a hand-port device. Hilar dissection and parenchymal transection were performed under pneumoperitoneum (*n* = 2) or through a mini-laparotomy incision (*n* = 7). The graft was extracted through the site of the hand-port device or the minilaparotomy. Operative time was 765 and 898 min in the laparoscopic RH patients, and it ranged from 310 to 575 min for the laparoscopy-assisted surgery. In one case, a fluid collection along the liver resection margin was reported, but it was resolved after percutaneous drainage.

At the Northwestern University, Baker et al. [67] retrospectively compared 33 open versus 33 laparoscopic living donor RHs, suggesting that laparoscopy could present equivalent safety, resource utilization, and effectiveness, with several adjunctive physical and psychological benefits. Donor operative times were shorter for the laparoscopic group (265 min versus 316 min). Blood loss and length of stay were comparable. Additionally, total hospitalization costs were equivalent. Finally, the group from Seoul compared single-port laparoscopy-assisted donor right hepatectomy (*n* = 40) with laparoscopy-assisted donor right hepatectomy (*n* = 20) and open donor right hepatectomy (*n* = 90); postoperative complication and reoperation rates revealed no significant differences, the single-port group showing the lowest level of postoperative pain [68]. 

Although very limited experiences have been reported worldwide until now, no mortalities have been encountered in laparoscopic living donor hepatectomy, whether adult or paediatric. Larger experience is needed in this field, but only centres with a coincident expertise in hepatic mini-invasive surgery and living donor LT should approach this type of surgery.

## 4. Laparoscopic-Assisted Liver Transplantation

The first nine cases of living donor LT through a short-midline incision combined with hand-assisted laparoscopic surgery have been reported in Japan [69]. All the patients were cirrhotic (median MELD score 14). Total hepatectomy was carried out through a hand-assisted laparoscopic approach with an 8 cm upper midline incision. Explantation of the diseased liver was obtained through the upper midline incision which was extended to 12 to 15 cm. Partial liver grafts were implanted through the upper midline incision. Median surgical time was 741 min, and the median blood loss was 3,940 g. 

This preliminary report of application of laparoscopy during LT procedure represents an extraordinary innovation, opening new porspectives in this fascinating surgical field. Further evolutions in the use of mini-invasive surgery in LT are expected in the next years. 

## 5. Laparoscopic Posttransplant Surgery

Postoperative laparoscopic management of LT patients is less common with respect to renal transplant recipients: in fact, laparoscopy is easily applied after kidney transplantation, given the fact that in this type of transplant the dissection is completely extraperitoneal.

In postLT patients, laparoscopy is a useful tool to solve a number of surgical complications; however, its use is strictly connected to surgeon's experience and versatility.

### 5.1. Laparoscopic Incisional Hernia Repair

Incisional hernia is caused by several aetiologies, many of whose could be concomitantly observed in LT recipients: advanced age, wound infection, ascites, steroids, diabetes, surgical techniques, suture material, retransplantation, bilateral sub-costal incision with midline extension, and, not less important, surgeon's experience. The most common site for incisional hernia in LT patients is located at the junction of the transverse and upper midline incisions [70]. In literature, the incidence of incisional hernia varies from 5% to 17% [71]. Large incisional and ventral hernias in nontransplant patients are now routinely repaired using laparoscopic technique. Laparoscopic ventral hernia repair seems to have a reduced risk of recurrence and infection compared to standard repair [72]. In LT patients, laparoscopic hernia repair is safe and with similar results when compared with open repair [70, 73]. 

Andreoni et al. [74] successfully completed 12 out of 13 attempted incisional hernia repairs by the laparoscopic technique in LT patients. Gore-Tex mesh was used. At the time of publication, they report no recurrence. They concluded that laparoscopic mesh repair of incisional hernias is practical and safe in patients with a surgical history of LT transplantation, with a low incidence of infections and no recurrence. However, in a monocentre study [74], a higher rate of postoperative seroma was observed in LT with respect to nontransplanted patients [75]. A study from Germany analyzed a population of 29 solid organs recipients: 15 cases were treated with intraperitoneal onlay mesh repair and 14 with conventional hernia repair [76]. Recurrence rate was 6% versus 50%, and complication rate was 33% versus 21% in laparoscopy and conventional groups, respectively. 

A study from Spain described 20 cases of laparoscopic incisional hernia repair in patients after LT, using a Bard Composix mesh, showing excellent results and few complications [77]. Observing the excellent results obtained using laparoscopy for the treatment of incisional hernia in LT patients, we can conclude that it could be safely performed also in this particular type of patients, and it could be considered a standard practice, mainly in an expert surgeon's hands. 

### 5.2. Other Indications

In the last years, different uses of laparoscopy have been attempted in LT recipients. Merenda et al. [78] reported two cases of intestinal occlusion caused by adhesions and three cases of lymphocele, all approached with laparoscopic surgery. In all cases but one, the authors were able to complete the surgery by laparoscopic means; in one of the two occlusions, the procedure was switched to laparotomy because of a choledochojejunal anastomosis lesion.

Gill et al. [79] reported a single case of a right adrenalectomy after LT in a 63-year-old female patient with a right adrenal mass and a previous story of left radical nephrectomy for a renal cell carcinoma and LT for primary biliary cirrhosis. A laparoscopic right adrenalectomy via the retroperitoneoscopic approach was successfully performed, and the patient was discharged home on the first postoperative day.

DeRoover and Sudan [80] documented the case of a 46-year-old female transplanted for primary sclerosing cholangitis who presented multiple splenic aneurysms and abdominal pain: after a laparoscopic splenectomy, the patient was discharged on postoperative day 3 free of symptoms. A Japanese experience [81] reported 5 cases of hand-assisted laparoscopic splenectomy for hypersplenism in living donor LT recipients. On the basis of the excellent results, the authors consider it as a possible standard procedure after LT.

Robles et al. [82] commented on 2 cases of biliary peritonitis after T-tube removal who failed conservative treatment and subsequently underwent laparoscopy: lysis of adhesions was carried out in the right upper quadrant, a Penrose drain was placed, and both patients were discharged home on postoperative day 4. In 2010, Zhu et al. [83] reported the first total laparoscopic hysterectomy after LT. Authors confirmed that no viscera adhesions were observed to the undersurface of the umbilicus.

In 2011, Lee et al. [84] were the first to successfully complete a laparoscopic total gastrectomy in a previously transplanted 72-year-old patient, showing that laparoscopy is a feasible method for gastric cancer treatment in LT patients.

Finally, the Hannover group reflected on the applicability of laparoscopy in the management of posttransplantation lymphoproliferative disorder in a pediatric population: 6 out of 34 (18%) solid organs recipients underwent laparoscopic biopsies because of the lack of superficial lesions, with a 83% success rate. In one patient, a trocar metastasis was identified and treated successfully with chemotherapy [85].

Despite few cases have been reported until now, we can affirm that several “conceptual” barriers in the field of laparoscopy have been overcome: previous LT no longer represents an absolute contraindication for laparoscopy; not only in case of small procedures (biopsies) or submesocolic and pelvic surgery, but also when supramesocolic organs are involved. Further experience is needed in this field and only surgeons with a high expertise in mini-invasive procedures must approach this type of surgery. However, the authors are confident that in the future laparoscopic or robotic surgery will substitute open surgery in many cases even in previously transplanted patients. 

## 6. Conclusion

Use of laparoscopy in the field of LT is safe and feasible. Mini-invasive approach is commonly adopted in the bridge treatment of HCC in patients waiting for LT: in case of LLS, laparoscopic procedure is recognized as the gold standard therapy. In living donor hepatectomy, and, recently, in LT, pure mini-invasive approaches or hybrid forms of laparoscopic and open surgery have been attempted. However, a limited number of reports are currently available on this subject, and great ability and confidence are recommended for starting these laparoscopy-assisted programs. Laparoscopy for abdominal surgery after LT has been demonstrated to be feasible and safe, not only in patients candidates for pelvic surgery, but also in case of surgery in the upper abdominal quadrants. In the next future, welcome improvement in technologies will give impulse to further expansion of this surgical area.

## References

[1] Cuschieri A, Dubois F, Mouiel J (1991). The European experience with laparoscopic cholecystectomy. *American Journal of Surgery*.

[2] Starzl TE, Marchioro TL, Vonkaulla KN, Hermann G, Brittain RS, Waddell WR (1963). Homotransplantation of the liver in humans. *Surgery, Gynecology & Obstetrics*.

[3] Calne RY, Thiru S, White DJG (1978). Cyclosporin A in patients receiving renal allografts from cadaver donors. *The Lancet*.

[4] Gagner M, Rheault M, Dubuc J (1992). Laparoscopic partial hepatectomy for liver tumor. *Surgical Endoscopy*.

[5] Azagra JS, Goergen M, Gilbart E, Jacobs D (1996). Laparoscopic anatomical (hepatic) left lateral segmentectomy—technical aspects. *Surgical Endoscopy*.

[6] Mala T, Edwin B, Rosseland AR, Gladhaug I, Fosse E, Mathisen Ø (2005). Laparoscopic liver resection: experience of 53 procedures at a single center. *Journal of Hepato-Biliary-Pancreatic Surgery*.

[7] Descottes B, Lachachi F, Sodji M (2000). Early experience with laparoscopic approach for solid liver tumors: initial 16 cases. *Annals of Surgery*.

[8] Cherqui D, Laurent A, Tayar C (2006). Laparoscopic liver resection for peripheral hepatocellular carcinoma in patients with chronic liver disease: midterm results and perspectives. *Annals of Surgery*.

[9] Biertho L, Waage A, Gagner M (2002). Laparoscopic hepatectomies. *Annales de Chirurgie*.

[10] Dagher I, Proske JM, Carloni A, Richa H, Tranchart H, Franco D (2007). Laparoscopic liver resection: results for 70 patients. *Surgical Endoscopy and Other Interventional Techniques*.

[11] Simillis C, Constantinides VA, Tekkis PP (2007). Laparoscopic versus open hepatic resections for benign and malignant neoplasms-a meta-analysis. *Surgery*.

[12] Ito K, Ito H, Are C (2009). Laparoscopic versus open liver resection: a matched-pair case control study. *Journal of Gastrointestinal Surgery*.

[13] Cai XJ, Yang J, Yu H (2008). Clinical study of laparoscopic versus open hepatectomy for malignant liver tumors. *Surgical Endoscopy and Other Interventional Techniques*.

[14] Endo Y, Ohta M, Sasaki A (2009). A comparative study of the long-term outcomes after laparoscopy-assisted and open left lateral hepatectomy for hepatocellular carcinoma. *Surgical Laparoscopy, Endoscopy and Percutaneous Techniques*.

[15] Topal B, Fieuws S, Aerts R, Vandeweyer H, Penninckx F (2008). Laparoscopic versus open liver resection of hepatic neoplasms: comparative analysis of short-term results. *Surgical Endoscopy and Other Interventional Techniques*.

[16] Dagher I, Di Giuro G, Dubrez J, Lainas P, Smadja C, Franco D (2009). Laparoscopic versus open right hepatectomy: a comparative study. *American Journal of Surgery*.

[17] Kim HH, Park EK, Seoung JS (2011). Liver resection for hepatocellular carcinoma: case-matched analysis of laparoscopic versus open resection. *Journal of the Korean Surgical Society*.

[18] Troisi RI, Van Huysse J, Berrevoet F (2011). Evolution of laparoscopic left lateral sectionectomy without the Pringle maneuver: through resection of benign and malignant tumors to living liver donation. *Surgical Endoscopy and Other Interventional Techniques*.

[19] Buell JF, Cherqui D, Geller DA (2009). The international position on laparoscopic liver surgery: the Louisville Statement, 2008. *Annals of Surgery*.

[20] Adam R, Azoulay D (2005). Is primary resection and salvage transplantation for hepatocellular carcinoma a reasonable strategy?. *Annals of Surgery*.

[21] Mazzaferro V, Todo S, Tzakis AG, Stieber AC, Makowka L, Starzl TE (1990). Liver transplantation in patients with previous portasystemic shunt. *American Journal of Surgery*.

[22] Steib A, Freys G, Lehmann C, Meyer C, Mahoudeau G (2001). Intraoperative blood losses and transfusion requirements during adult liver transplantation remain difficult to predict. *Canadian Journal of Anesthesia*.

[23] Laurent A, Tayar C, Andréoletti M, Lauzet JY, Merle JC, Cherqui D (2009). Laparoscopic liver resection facilitates salvage liver transplantation for hepatocellular carcinoma. *Journal of Hepato-Biliary-Pancreatic Surgery*.

[24] Lai ECH, Tang CN, Ha JPY, Li MKW (2009). Laparoscopic liver resection for hepatocellular carcinoma ten-year experience in a single center. *Archives of Surgery*.

[25] Dagher I, Lainas P, Carloni A (2008). Laparoscopic liver resection for hepatocellular carcinoma. *Surgical Endoscopy and Other Interventional Techniques*.

[26] Aldrighetti L, Guzzetti E, Pulitanò C (2010). Case-matched analysis of totally laparoscopic versus open liver resection for HCC: short and middle term results. *Journal of Surgical Oncology*.

[27] Tranchart H, Di Giuro G, Lainas P (2010). Laparoscopic resection for hepatocellular carcinoma: a matched-pair comparative study. *Surgical Endoscopy and Other Interventional Techniques*.

[28] Belli G, Limongelli P, Fantini C (2009). Laparoscopic and open treatment of hepatocellular carcinoma in patients with cirrhosis. *British Journal of Surgery*.

[29] Hu BS, Chen K, Tan HM (2011). Comparison of laparoscopic vs open liver lobectomy (segmentectomy) for hepatocellular carcinoma. *World Journal of Gastroenterology*.

[30] Panaro F, Piardi T, Cag M (2011). Robotic liver resection as a bridge to liver transplantation. *Journal of the Society of Laparoendoscopic Surgeons*.

[31] Lai ECH, Tang CN, Yang GPC, Li MKW (2011). Multimodality laparoscopic liver resection for hepatic malignancy—from conventional total laparoscopic approach to robot-assisted laparoscopic approach. *International Journal of Surgery*.

[32] Choi SB, Park JS, Kim JK (2008). Early experiences of robotic-assisted laparoscopic liver resection. *Yonsei Medical Journal*.

[33] Asahina Y, Nakanishi H, Izumi N (2009). Laparoscopic radiofrequency ablation for hepatocellular carcinoma: review. *Digestive Endoscopy*.

[34] Siperstein A, Garland A, Engle K (2000). Laparoscopic radiofrequency ablation of primary and metastatic liver tumors: technical considerations. *Surgical Endoscopy*.

[35] Panaro F, Piardi T, Audet M (2010). Laparoscopic ultrasound-guided radiofrequency ablation as a bridge to liver transplantation for hepatocellular carcinoma: preliminary results. *Transplantation Proceedings*.

[36] Tandan VR, Asch M, Margolis M, Page A, Gallinger S (1997). Laparoscopic vs. open intraoperative ultrasound examination of the liver: a controlled study. *Journal of Gastrointestinal Surgery*.

[37] Montorsi M, Santambrogio R, Bianchi P (2001). Laparoscopy with laparoscopic ultrasound for pretreatment staging of hepatocellular carcinoma: a prospective study. *Journal of Gastrointestinal Surgery*.

[38] Kokudo N, Bandai Y, Imanishi H (1996). Management of new hepatic nodules detected by intraoperative ultrasonography during hepatic resection for hepatocellular carcinoma. *Surgery*.

[39] Takigawa Y, Sugawara Y, Yamamoto J (2001). New lesions detected by intraoperative ultrasound during liver resection for hepatocellular carcinoma. *Ultrasound in Medicine and Biology*.

[40] Torzilli G, Makuuchi M (2003). Intraoperative ultrasonography in liver cancer. *Surgical Oncology Clinics of North America*.

[41] Cerwenka H, Raith J, Bacher H (2003). Is intraoperative ultrasonography during partial hepatectomy still necessary in the age of magnetic resonance imaging?. *Hepato-Gastroenterology*.

[42] Zhang K, Kokudo N, Hasegawa K (2007). Detection of new tumors by intraoperative ultrasonography during repeated hepatic resections for hepatocellular carcinoma. *Archives of Surgery*.

[43] Santambrogio R, Opocher E, Zuin M (2009). Surgical resection versus laparoscopic radiofrequency ablation in patients with hepatocellular carcinoma and child-pugh class a liver cirrhosis. *Annals of Surgical Oncology*.

[44] Tanabe KK, Curley SA, Dodd GD, Siperstein AE, Goldberg SN (2004). Radiofrequency ablation: the experts weigh in. *Cancer*.

[45] Livraghi T, Solbiati L, Meloni MF, Gazelle GS, Halpern EF, Goldberg SN (2003). Treatment of focal liver tumors with percutaneous radio-frequency ablation: complications encountered in a multicenter study. *Radiology*.

[46] Casaril A, Abu Hilal M, Harb A, Campagnaro T, Mansueto G, Nicoli N (2008). The safety of radiofrequency thermal ablation in the treatment of liver malignancies. *European Journal of Surgical Oncology*.

[47] Yun KC, Rhim H, Yong SA, Mi YK, Hyo KL (2006). Percutaneous radiofrequency ablation therapy of hepatocellular carcinoma using multitined expandable electrodes: comparison of subcapsular and nonsubcapsular tumors. *American Journal of Roentgenology*.

[48] Esteves E, Neto EC, Neto MO, Devanir J, Pereira RE (2002). Laparoscopic Kasai portoenterostomy for biliary atresia. *Pediatric Surgery International*.

[49] Martinez-Ferro M, Esteves E, Laje P (2005). Laparoscopic treatment of biliary atresia and choledochal cyst. *Seminars in Pediatric Surgery*.

[50] Ure BM, Kuebler JF, Schukfeh N, Engelmann C, Dingemann J, Petersen C (2011). Survival with the native liver after laparoscopic versus conventional kasai portoenterostomy in infants with biliary atresia: a prospective trial. *Annals of Surgery*.

[51] Chan KWE, Lee KH, Mou JWC, Cheung STG, Tam YHP (2011). The outcome of laparoscopic portoenterostomy for biliary atresia in children. *Pediatric Surgery International*.

[52] Flowers JL, Jacobs S, Cho E (1997). Comparison of open and laparoscopic live donor nephrectomy. *Annals of Surgery*.

[53] Ratner LE, Kavoussi LR, Sroka M (1997). Laparoscopic assisted live donor nephrectomy—a comparison with the open approach. *Transplantation*.

[54] Ratner LE, Hiller J, Sroka M (1997). Laparoscopic live donor nephrectomy removes disincentives to live donation. *Transplantation Proceedings*.

[55] Intaraprasong P, Sobhonslidsuk A, Tongprasert S (2010). Donor outcomes after Living Donor Liver Transplantation (LDLT). *Journal of the Medical Association of Thailand*.

[56] Cherqui D, Soubrane O, Husson E (2002). Laparoscopic living donor hepatectomy for liver transplantation in children. *The Lancet*.

[57] Lin E, Gonzalez R, Venkatesh KR (2003). Can current technology be integrated to facilitate laparoscopic living donor hepatectomy?. *Surgical Endoscopy and Other Interventional Techniques*.

[58] Soubrane O, Cherqui D, Scatton O (2006). Laparoscopic left lateral sectionectomy in living donors: safety and reproducibility of the technique in a single center. *Annals of Surgery*.

[59] Troisi R, Debruyne R, Rogiers X (2009). Laparoscopic living donor hepatectomy for pediatric liver transplantation. *Acta Chirurgica Belgica*.

[60] Coelho JCU, de Freitas ACT, Mathias JEF (2009). Laparoscopic resection of the left lateral segment of the liver in living donor liver transplantation. *Revista do Colegio Brasileiro de Cirurgioes*.

[61] Kim KH, Jung DH, Park KM (2011). Comparison of open and laparoscopic live donor left lateral sectionectomy. *British Journal of Surgery*.

[62] Thenappan A, Jha RC, Fishbein T (2011). Liver allograft outcomes after laparoscopic-assisted and minimal access live donor hepatectomy for transplantation. *American Journal of Surgery*.

[63] Koffron AJ, Kung R, Baker T, Fryer J, Clark L, Abecassis M (2006). Laparoscopic-assisted right lobe donor hepatectomy. *American Journal of Transplantation*.

[64] Kurosaki I, Yamamoto S, Kitami C (2006). Video-assisted living donor hemihepatectomy through a 12-cm incision for adult-to-adult liver transplantation. *Surgery*.

[65] Suh KS, Yi NJ, Kim J (2008). Laparoscopic hepatectomy for a modified right graft in adult-to-adult living donor liver transplantation. *Transplantation Proceedings*.

[66] Suh KS, Yi NJ, Kim T (2009). Laparoscopy-assisted donor right hepatectomy using a hand port system preserving the middle hepatic vein branches. *World Journal of Surgery*.

[67] Baker TB, Jay CL, Ladner DP (2009). Laparoscopy-assisted and open living donor right hepatectomy: a comparative study of outcomes. *Surgery*.

[68] Choi HJ, You YK, Na GH (2012). Single-port laparoscopy-assisted donor right hepatectomy in living donor liver transplantation: sensible approach or unnecessary hindrance?. *Transplantation Proceedings*.

[69] Eguchi S, Takatsuki M, Soyama A (2011). Elective living donor liver transplantation by hybrid hand-assisted laparoscopic surgery and short upper midline laparotomy. *Surgery*.

[70] Kurmann A, Beldi G, Vorburger SA, Seiler CA, Candinas D (2010). Laparoscopic incisional hernia repair is feasible and safe after liver transplantation. *Surgical Endoscopy and Other Interventional Techniques*.

[71] Piardi T, Audet M, Panaro F (2010). Incisional hernia repair after liver transplantation: role of the mesh. *Transplantation Proceedings*.

[72] Heniford BT, Park A, Ramshaw BJ, Voeller G, Hunter JG, Fitzgibbons RJ (2003). Laparoscopic repair of ventral hernias: nine years’ experience with 850 consecutive hernias. *Annals of Surgery*.

[73] Mekeel K, Mulligan D, Reddy KS, Moss A, Harold K (2007). Laparoscopic incisional hernia repair after liver transplantation. *Liver Transplantation*.

[74] Andreoni KA, Lightfoot H, Gerber DA, Johnson MW, Fair JH (2002). Laparoscopic incisional hernia repair in liver transplant and other immunosuppressed patients. *American Journal of Transplantation*.

[75] Harold K, Mekeel K, Spitler J (2009). Outcomes analysis of laparoscopic ventral hernia repair in transplant patients. *Surgical Endoscopy and Other Interventional Techniques*.

[76] Scheuerlein H, Rauchfuss F, Gharbi A, Heise M, Settmacher U (2011). Laparoscopic incisional hernia repair after solid-organ transplantation. *Transplantation Proceedings*.

[77] Gianchandani R, Moneva E, Marrero P (2011). Feasibility and effectiveness of laparoscopic incisional hernia repair after liver transplantation. *Transplantation Proceedings*.

[78] Merenda R, Gerunda GE, Neri D (2000). Laparoscopic surgery after orthotopic liver transplantation. *Liver Transplantation*.

[79] Gill IS, Meraney AM, Mayes JT, Bravo EL (2001). Laparoscopic right adrenalectomy after liver transplantation. *Transplantation*.

[80] DeRoover A, Sudan D (2001). Treatment of multiple aneurysms of the splenic artery after liver transplantation by percutaneous embolization and laparoscopic splenectomy. *Transplantation*.

[81] Uehara H, Kawanaka H, Akahoshi T (2009). The feasibility and effectiveness of a hand-assisted laparoscopic splenectomy for hypersplenism in patients after living-donor liver transplantation. *Surgical Laparoscopy, Endoscopy and Percutaneous Techniques*.

[82] Robles R, Parrilla P, Lujan JA, Torralba JA, Ramirez P, Bueno FS (1997). Laparoscopic treatment of biliary peritonitis after T tube removal in patients undergoing orthotopic liver transplantation. *British Journal of Surgery*.

[83] Zhu HB, Jin Y, Xu ST, Xia YX, Xie LP (2010). Total laparoscopic hysterectomy after liver transplantation. *Hepatobiliary and Pancreatic Diseases International*.

[84] Lee MS, Kim EY, Lee JH (2011). Laparoscopy-assisted distal gastrectomy for gastric cancer after liver transplantation. *Journal of the Korean Surgical Society*.

[85] Metzelder ML, Schober T, Grigull L (2011). The role of laparoscopic techniques in children with suspected post-transplantation lymphoproliferative disorders.. *Journal of Laparoendoscopic & Advanced Surgical Techniques*.

